# Goal-directed hemodynamic resuscitation in high-risk patients undergoing cardiac surgery: a randomized controlled trial - preliminary data (GRICCS STUDY)

**DOI:** 10.1186/cc10804

**Published:** 2012-03-20

**Authors:** E Osawa, A Rhodes, J Fukushima, J Almeida, F Jatene, R Nakamura, M Sundin, J Auler, R Kalil Filho, F Galas, L Hajjar

**Affiliations:** 1 Heart Institute, São Paulo, Brazil; 2 Charing Cross Hospital, Imperial College NHS Trust, London, UK

## Introduction

Low cardiac output is a frequent clinical circumstance after cardiac surgery and results in higher morbidity and mortality rates. Goal-directed therapy (GDT) is a validated design that has been proved to reduce the number of perioperative outcomes. We investigated the results of a cardiac index optimization protocol through the use of the LiDCO rapid device.

## Methods

A prospective study that randomized 34 high-risk patients (EuroSCORE higher than 6 or LVEF lower than 45%) to a GDT protocol or a conventional hemodynamic therapy. Patients from the GDT group were resuscitated to a cardiac index higher than 3 l/minute/m^2 ^through the implementation of a three-step approach: (1) fluid challenge of 250 ml aliquots, (2) dobutamine infusion up to a dose of 20 μg/kg/minute, and (3) blood transfusion to reach a hematocrit higher than 28%. The control group was managed according to institutional protocol. Categorical variables were compared using Fisher's exact test and categorical variables were compared using the Mann-Whitney U test.

## Results

Sixteen patients from the GDT group were compared with 18 patients from the control group. There was a tendency towards reduction in ICU stay in patients from GDT group in relation to the control group (7 days vs. 6 days, *P *= 0.18). Comparison of the primary endpoint variable (composite of death or major postoperative complications within 30 days after surgery or before discharge) between groups showed a reduced complication rate in the GDT group (52.2% vs. 45.6%, *P *= 0.12), mainly attributed to worse acute renal failure RIFLE criteria in the control group.

## Conclusion

Goal-directed hemodynamic resuscitation with the use of a minimally invasive device seems to be a promising perioperative strategy aimed at reducing the rates of worse outcomes and the ICU stay after cardiac surgery.

